# Axillary evaluation is not warranted in patients preoperatively diagnosed with ductal carcinoma in situ by core needle biopsy

**DOI:** 10.1002/cam4.2623

**Published:** 2019-10-29

**Authors:** Jing Si, Rong Guo, Naisi Huang, Bingqiu Xiu, Qi Zhang, Weiru Chi, Jiong Wu

**Affiliations:** ^1^ Department of Breast Surgery Fudan University Shanghai Cancer Center Shanghai China; ^2^ Collaborative Innovation Center for Cancer Medicine Shanghai China

**Keywords:** axillary evaluation, core needle biopsy, ductal carcinoma in situ

## Abstract

**Background:**

Patients diagnosed with ductal carcinoma in situ (DCIS) by core needle biopsy (CNB) have a great chance of upstaging to invasive cancer. Positive axillary status can be found in these patients. This study sought to identify clinicopathological factors associated with upstaging and axillary metastasis in patients preoperatively diagnosed with DCIS by CNB.

**Materials and Methods:**

This study identified 604 patients (cT1‐3N0M0) with preoperative diagnosis of pure DCIS by CNB who had undergone axillary evaluation from August 2006 to December 2015 at Fudan University Shanghai Cancer Center (FUSCC). Predictors of upstaging and axillary lymph nodes metastasis were analyzed, respectively.

**Results:**

Of all 604 patients, 121 (20.03%) and 193 (31.95%) patients were upstaged to DCIS with microinvasion (DCISM) and invasive breast cancer (IBC). Positive axillary lymph nodes were identified in 41 (6.79%) patients. Predictors of upstaging included tumor size on ultrasonography (>2 cm) (OR 1.786, *P* = .002) and ER+HER2+ status (OR 1.874, *P* = .022) in multivariate analysis. Factors associated with axillary lymph nodes metastasis included tumor size on pathology (OR 2.336, *P* = .038) and number of lesions (OR 3.354, *P* = .039) in multivariate analysis. In addition, upstaging on final pathology had a significant influence on axillary lymph nodes status (*P* < .001).

**Conclusion:**

Axillary evaluation was recommended in patients with larger tumor size (>2 cm), multifocal lesions or ER+HER2+ status. Despite of a 51.98% upstaging rate, the rate of axillary metastasis in these patients was relatively low, supporting the omission of axillary evaluation in selected patients with low risk of upstaging or axillary metastasis.

## Graphical abstract

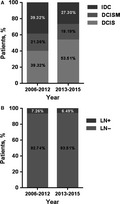

## 1 INTRODUCTION

Core needle biopsy (CNB) has become a standard method for breast cancer diagnosis, due to the avoidance of more invasive biopsies. However, the natural limitation in the volume of sampling of this biopsy method can result in underestimation. For example, patients diagnosed with ductal carcinoma in situ (DCIS) by CNB can be upstaged to DCIS with microinvasion (DCISM) or invasive breast cancer (IBC).^1^, ^2^ According to previous studies, approximately 26% of patients diagnosed with DCIS were upstaged to invasive disease on the final pathological diagnosis, with an overall range 8.8%‐51.5%.^3^, ^4^, ^5^, ^6^, ^7^, ^8^, ^9^, ^10^, ^11^

With the presence of upstaging in patients preoperatively diagnosed with DCIS by CNB, a major concern for surgeons is whether to evaluate the ALNs at the primary operation. Both National Comprehensive Cancer Network (NCCN) and American Society of Clinical Oncology (ASCO) guidelines recommended that sentinel lymph node biopsy (SLNB) should be performed in patients for whom axillary evaluation is difficult in stage two operation, such as patients who received mastectomy.^12^, ^13^ Also, patients highly suspicious for IBC either with palpable mass or high‐risk images in mammography should receive axillary evaluation in case of upstaging in ALNs status.^9^, ^12^, ^13^, ^14^

In this study, we identified clinicopathological factors associated with upstaging and ALNs metastasis in patients preoperatively diagnosed with DCIS by CNB. The aim of this study was to identify whether axillary evaluation can be omitted in these patients.

## 2 METHODS

### 2.1 Patients

We retrospectively reviewed the medical records of cN0 patients preoperatively diagnosed with pure DCIS by CNB who underwent axillary evaluation between August 2006 and December 2015 at Fudan University Shanghai Cancer Center (FUSCC). Patients were excluded if: (a) patients were male; (b) neoadjuvant chemotherapy was received prior to surgery; (c) patients had bilateral breast cancer; or (d) patients had a history of breast cancer. The study was approved by the Ethics Committee of FUSCC.

### 2.2 Surgical procedures and axillary evaluation

All patients underwent histological diagnosis preoperatively via a 14‐gauge core needle biopsy. Patients who were inclusive underwent the primary operation in our institution, including mastectomy and breast‐conserving surgery (BCS). The final pathological findings were classified as pure DCIS, DCISM, and IBC. Microinvasion was defined as invasive portion no more than 1mm. Immunostaining for estrogen receptor (ER) and progesteron receptor (PR) was performed and cases with 1% or more positive staining were considered as positive staining. Human epidermal growth factor receptor 2 (HER2) positivity was defined as those cases where immunohistochemistry (IHC) staining was 3+ alone or 2+ with fluorescence in situ hybridization (FISH) positivity.

In this study, SLNB was performed at the same time as the breast surgery. Histological assessment with hematoxylin‐eosin staining performed postoperatively served as the golden standard. A positive SLN was defined as the presence of either micrometastasis (>200 cells or >0.2 mm, but <2.0 mm) or macrometastasis (>2.0 mm) identified on hematoxylin‐eosin staining. Patients with intraoperatively positive SLNs were required to undergo axillary lymph nodes dissection (ALND). Level I and level II ALND was performed according to a standard ALND procedure.

### 2.3 Statistical analysis

The clinicopathological variables were compared between pure DCIS group and upstaging group according to the final pathological findings using Chi‐square test for categorical variables. Also, variables were compared between the axillary metastasis group and the axillary nonmetastasis group. Multivariate logistic regression analyses were performed to investigate the risk predictors of upstaging and axillary metastasis. Two‐tailed *P* values were adopted, and *P* < .05 was considered significant. All statistical analyses were performed using SPSS statistical software version 17.0 (IBM).

## 3 RESULTS

### 3.1 Baseline characteristics

A total of 604 patients met the criteria. The average age of these patients was 51.00 years (range 24‐83 years). Clinicopathological characteristics of the entire cohort are shown in Table 1. At the initial presentation of breast cancer, 526 (87.09%) patients presented with lumps, with an average diameter of 24.68 mm (range 10‐79 mm). The majority (548, 90.73%) underwent mastectomy, and 56 (9.27%) patients chose BCS. Overall, 121 (20.03%) and 193 (31.95%) patients were upstaged to DCISM and IBC on final pathology, respectively. Several patients with combined pathological type were detected, divided by final pathology (Table 2). After examining axillary status in these patients, we found that patients combined with invasive micropapillary carcinoma in IBC group were more likely to have positive ALNs (66.67% vs 15.51%, *P* < .001).

**Table 1:** Baseline clinicopathological characteristics of patients preoperatively diagnosed with DCIS by CNB

Variables	Total N = 604	%
Age		
≤50	309	51.16
>50	295	48.84
Menopause		
No	297	49.17
Yes	282	46.69
Unknown	25	4.14
BC family history		
No	481	79.64
Yes	123	20.36
BMI		
<25	457	75.66
≥25	126	20.86
Unknown	21	3.48
Tumor size on ultrasonography		
cT1	229	37.91
cT2‐3	331	54.80
Unknown	44	7.28
Calcification on mammography		
Yes	363	60.10
No	79	13.08
Unknown	162	26.82
MRI		
No	248	41.06
Yes	356	58.94
Quadrate		
Upper outer	220	36.42
Others	384	63.58
Surgical methods		
Mastectomy	548	90.73
BCS	56	9.27
Axillary evaluation		
SLNB	513	84.93
ALND	91	15.07
Histological grade		
Non‐high	299	49.50
High	253	41.89
Unknown	52	8.61
Tumor size on pathology		
≤2 cm	208	34.44
>2 cm	396	65.56
Number of lesions		
Unifocal	581	96.19
Multifocal	23	3.81
Ki67		
≤14%	136	22.51
>14%	418	69.20
Unknown	50	8.29
Molecular type		
ER+HER2−	227	37.58
ER+HER+	113	18.71
ER−HER2+	200	33.11
ER−HER2−	54	8.94
Unknown	10	1.66

Abbreviations: ALND, axillary lymph node dissection; BC, breast cancer; BCS, breast‐conserving surgery; BMI, body mass index; CNB, core needle biopsy; DCIS, ductal carcinoma in situ; ER, estrogen receptor; HER2, human epidermal growth factor receptor 2; MRI, magnetic resonance imaging; SLNB, sentinel lymph node biopsy.

**Table 2:** Final pathology in patients with combined pathological type

Specific combined pathological type	DCIS (N = 290) n (%)	DCISM (N = 121) n (%)	IBC (N = 193) n (%)
Other carcinoma in situ^a^	20 (6.90)	3 (2.48)	3 (1.55)
Other invasive type^b^	0 (0)	0 (0)	14 (7.25)
Sclerosing adenosis	13 (4.48)	4 (3.31)	0 (0)
Necrosis	7 (2.41)	3 (2.48)	0 (0)
Paget's disease in nipple	4 (1.38)	2 (1.65)	3 (1.55)

Abbreviations: DCIS, ductal carcinoma in situ; DCISM, ductal carcinoma in situ with microinvasion; IBC, invasive breast cancer.

a Solid papillary carcinoma and encapsulated papillary carcinoma.

b Invasive lobular carcinoma, invasive papillary carcinoma, invasive micropapillary carcinoma, invasive neuroendocrine carcinoma.

**Figure 1:**
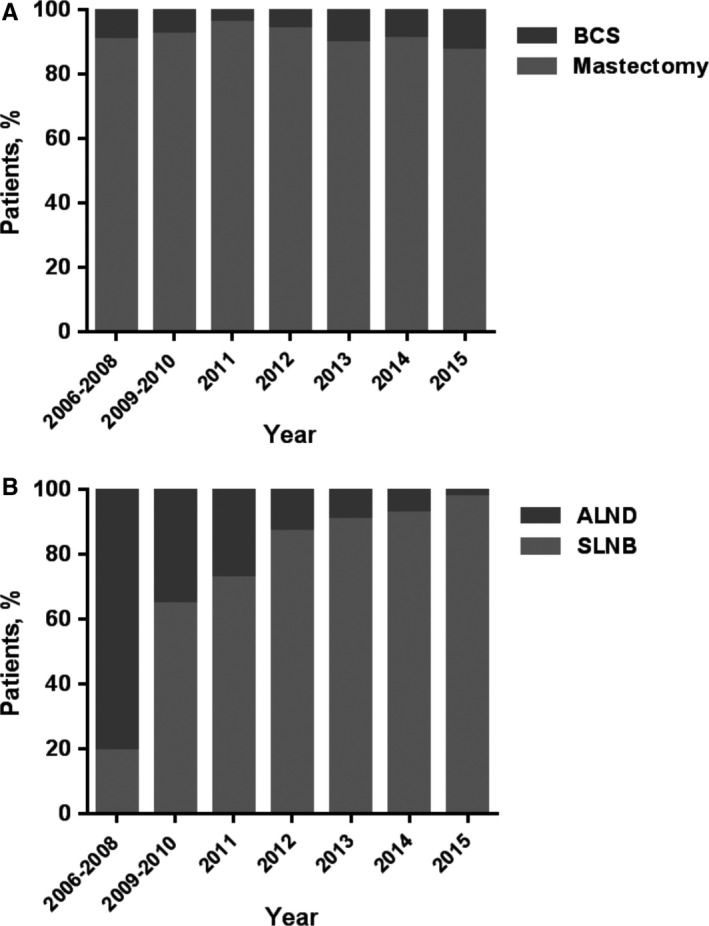
Trends in surgical options (A) and axillary evaluation (B) of patients preoperatively diagnosed with pure ductal carcinoma in situ by core needle biopsy at FUSCC from 2006 to 2015

**Figure 2:**
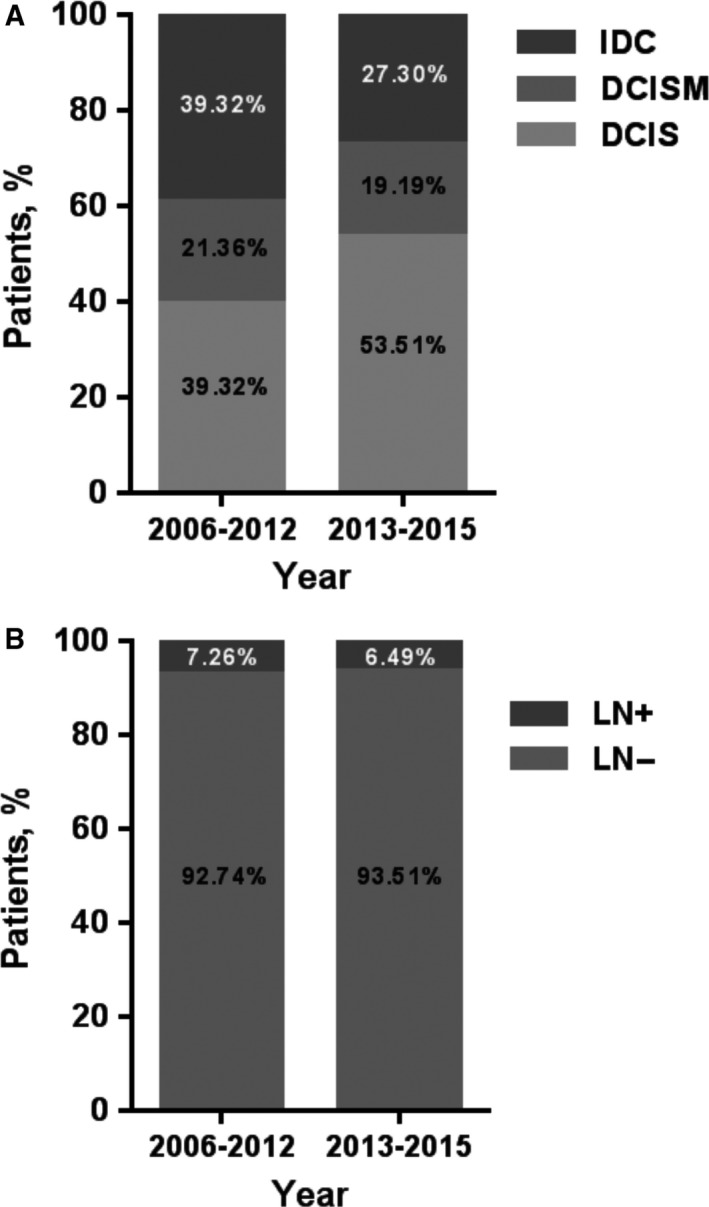
Comparison of proportion of upstaging (A) and axillary metastasis (B) in patients preoperatively diagnosed with pure ductal carcinoma in situ by core needle biopsy between 2006–2012 and 2013–2015

The trends in surgical options have hardly altered over time, whereas the paradigm of axillary evaluation has changed (Figure 1A,B). SLNB was introduced at our institution in 2006 and was performed by only a few surgeons in the first few years. Before 2012, nearly one third of patients in average underwent ALND, however, the percentile significantly decreased to 2.60% in 2015 (Table 3). Because of the increase in the prevalence of SLNB, we divided the whole cohort according to time periods: 2006‐2012 and 2013‐2015. We further analyzed upstaging and axillary metastasis in these two periods. We found that the proportion of upstaging decreased from 60.68% to 46.49% (*P* = .002; Figure 2A), which might because of the increase in preoperatively MRI (*P* < .001). While, the rate of axillary metastasis had no significant difference in these two periods (Figure 2B).

**Table 3:** Trends in surgical options and axillary evaluation from 2006 to 2015 in FUSCC

Year	Total	Mastectomy	%	BCS	%	SLNB	%	ALND	%
2006‐2008	21	19	90.48	2	9.52	4	19.05	17	80.95
2009‐2010	62	57	91.94	5	8.06	40	64.52	22	35.48
2011	69	66	95.65	3	4.35	50	72.46	19	27.54
2012	82	77	93.90	5	6.10	71	86.59	11	13.41
2013	95	85	89.47	10	10.53	86	90.53	9	9.47
2014	121	110	90.91	11	9.09	112	92.56	9	7.44
2015	154	134	87.01	20	12.99	150	97.40	4	2.60
Total	604	548	90.73	56	9.27	513	84.93	91	15.07

Abbreviations: ALND, axillary lymph node dissection; BCS, breast‐conserving surgery; SLNB, sentinel lymph node biopsy.

### 3.2 Axillary lymph nodes status

Of all patients, 513 (84.93%) underwent SLNB and 91 (15.07%) underwent ALND. Positive ALNs were identified in 41 (6.79%) patients based on the final paraffin section pathology, of which 85.37% (35/41) had one to two positive ALNs, 14.63% (6/41) had three or more positive ALNs. Among the patients with positive ALNs, 30 patients received SLNB: four patients with isolated tumor cells, six patients with micrometastasis, and 20 patients with macrometastasis.

Of the 513 patients who received SLNB, 30 of them had positive SLNs. Of these 30 patients, 22 of them received further axillary evaluation, and three showed positive in ALND. All three patients were IBC patients. The rate of axillary node positivity after a positive sentinel node was 10.00% (3/30) (Table 4). We further found that the finding of positive ALNs was correlated with the extent of invasion at the final pathology. Positive ALNs occurred in 1.38% (4/290) of the patients with pure DCIS, 3.31% (4/121) of the patients with microinvasion, and 17.10% (33/193) of the patients with IBC (*P* < .001). However, this correlation was not shown between patients with DCIS and DCISM in pairwise comparison (*P* = .198).

**Table 4:** Correlation of the rate of positive SLNs and the rate of positive nodes after ALND for patients in different groups according to their final pathology

	Patients received SLNB N = 513	Positive SLNs N = 30	Further received ALND N = 22	Positive ALND N = 3
DCIS	261	4	3	0
DCISM	103	4	2	0
IBC	149	22	17	3

### 3.3 Predictors of upstaging on final pathology

Various clinicopathological factors were examined in order to determine predictors of upstaging on final pathology (Table 5). Compared with patients in DCIS group, patients with upstaging tend to have larger tumor size on ultrasonography (61.15% vs 47.93%, *P* = .005) and higher Ki67 level (72.61% vs 65.52%, *P* = .046). Also, patients underwent mastectomy or patients with multifocal lesions had the trends of being upstaged, which did not reach significant differences. The two groups were comparable in age (*P* = .230), breast cancer family history (*P* = .229), body mass index (BMI) (*P* = .881), lesion classification (*P* = .169), and tumor position (*P* = .656), whereas they differed in the profile of molecular subtype (*P* = .007). Predictors with *P* < .05 in the univariate analysis were included in a multivariate logistic regression analysis, which showed that patients with larger tumor size on ultrasonography (>2.0 cm) (OR 1.786, 95% CI 1.237‐2.580, *P* = .002) were more likely to be upstaged on final pathology. Also, ER+HER2+ patients were more likely to be upstaged than ER+HER2− patients (OR 1.874, 95% CI 1.095‐3.206, *P* = .022).

**Table 5:** Univariate and multivariate analysis of predictors of upstaging on final pathology

Variables	DCIS N = 290	%	DCISM&IBC N = 314	%	Univariate *P*‐value	Multivariate OR (95% CI), *P*‐value
Tumor size on ultrasonography					**.005**	
cT1	127	43.79	102	32.48		Ref
cT2‐3	139	47.93	192	61.15		1.786 (1.237‐2.580), **.002**
Unknown	24	8.28	20	6.37		
Ki67					**.046**	
≤14%	78	26.90	58	18.47		Ref
>14%	190	65.52	228	72.61		1.547 (0.989‐2.421), .056
Unknown	22	7.59	28	8.92		
Molecular type					**.007**	
ER+HER2−	116	40.00	111	35.35		Ref
ER+HER+	39	13.45	74	23.57		1.874 (1.095‐3.206), **.022**
ER−HER2+	106	36.55	94	29.94		0.753 (0.479‐1.184), .220
ER−HER2‐	22	7.59	32	10.19		1.703 (0.877‐3.305), .116
Unknown	7	2.41	3	0.96		

Abbreviations: CI, confidence interval; DCIS, ductal carcinoma in situ; DCISM, ductal carcinoma in situ with microinvasion; ER, estrogen receptor; HER2, human epidermal growth factor receptor 2; IBC, invasive breast cancer; OR, odd ratio.

Bold indicates significant *P*‐values (*P* < .05).

### 3.4 Factors associated with axillary lymph nodes metastasis

Univariate and multivariate analysis were performed in order to investigate factors associated with positive ALNs (Table 6). In univariate analysis, compared with patients in ALN− group, patients in ALN+ group were more likely to have larger tumor size on pathology (>2 cm) (80.49% vs 64.48%, *P* = .037) and multifocal lesions (9.76% vs 3.37%, *P* = .039). Both factors reached statistical significance in multivariate analysis. Patients with larger tumor size on pathology (>2 cm) and multifocal lesions had 2.336‐fold and 3.354‐fold greater risks of axillary metastasis, respectively (95% CI 1.047‐5.213, *P* = .038; 95% CI 1.065‐10.564, *P* = .039).

**Table 6:** Univariate and multivariate analysis of factors associated with axillary lymph nodes status

Variables	ALN+ N = 41	%	ALN‐ N = 563	%	Univariate *P*‐value	Multivariate OR (95% CI), *P*‐value
Tumor size on pathology					**.037**	
≤2 cm	8	19.51	200	35.52		Ref
>2 cm	33	80.49	363	64.48		2.336 (1.047‐5.213), **.038**
Number of lesions					**.039**	
Unifocal	37	90.24	544	96.63		Ref
Multifocal	4	9.76	19	3.37		3.354 (1.065‐10.564), **.039**

Abbreviations: ALNs, axillary lymph nodes; CI, confidence interval; OR, odd ratio.

Bold indicates significant *P*‐values (*P* < .05).

## 4 DISCUSSION

This study has investigated the factors associated with DCIS upstaging and axillary metastasis. The rate of upstaging to DCISM and IBC was 20.03% and 31.95%, respectively. Independent predictors of upstaging included larger tumor size on ultrasonography (>2 cm) and molecular subtype (ER+HER2+). Axillary metastasis rate was 6.79%. Factors associated with positive ALNs were larger tumor size on pathology (>2 cm) and multifocal lesions.

In previous studies, approximately 26% of patients diagnosed with DCIS was upstaged to IBC (range 8.8%‐51.5%).^3^, ^4^, ^5^, ^6^, ^7^, ^8^, ^9^, ^10^, ^11^ And the rate of upstaging to DCISM ranged 4%‐29.6%.^15^, ^16^, ^17^ In this study, we have reported a relatively high rate of underestimation, 51.98% in total without subgrouping either upstaging to microinvasion or IBC. This variable proportion of upstaging could be a result of pathologists’ interpretation factors.^18^ Our current findings on independent predictors of upstaging are consistent with previous reports. A meta‐analysis reported by Brennan M E et al, which included 7350 cases of DCIS diagnosed by CNB, showed that tumor size was one of the strongest independent predictors of underestimation.^18^ In numerous previous studies, large tumor size, palpable lump, and number of lesions were associated with the risk of upstaging.^2^, ^8^, ^15^, ^18^, ^19^, ^20^, ^21^ Other factors, such as nuclear grade, comedo necrosis, sclerosing adenosis, and CNB method, were also correlated with upstaging.^2^, ^8^, ^18^, ^19^, ^20^ Studies on molecular subtype as a predictor of upstaging are rare.^8^, ^19^, ^21^, ^22^ Some studies showed a correlation between negative hormone receptor (HR) and invasion of DCIS,^8^, ^21^ and others showed that positive HER2 status was associated with upstaging.^19^, ^22^ In this study, we have identified several independent predictors of underestimation, and larger tumor size on ultrasonography (>2 cm) was the strongest.

In our study, the rate of positive ALNs was 6.79%, which was comparable with a previous meta‐analysis reported by Ansari B et al, showing that the axillary metastasis rate was 7.4% in patients with DCIS diagnosed by CNB.^23^ Also, we identified larger tumor size and multifocal lesions as factors associated with axillary metastasis. Theoretically, DCIS is defined on the basis that the cancer has not broken through the basement membrane of the breast duct, which means it does not have the potential to metastasize, thus, no axillary evaluation is needed. While, with the major issue of upstaging in preoperative DCIS diagnosis, whether these patients need axillary evaluation is controversial due to the risk of axillary metastasis. Previous data showed, the overall axillary metastasis rate was 5% in DCIS patients, which was most likely due to underlying invasive carcinoma, however, it increased to 10%‐20% if preoperatively underestimated was proved on final pathology.^2^, ^10^, ^24^, ^25^, ^26^ In this study, 1.38% and 3.31% of patients had positive ALNs in DCIS and DCISM group, separately, which showed no significant difference. However, 17.10% of patients with IBC showed axillary metastasis. Thus, when performing axillary evaluation at the primary operation, surgeons are faced with the dilemma of avoiding second operation if the final pathology upstaged, or performing an unnecessary procedure in pure DCIS patients.

Routine axillary evaluation in all patients diagnosed with DCIS preoperatively is not justified, due to the low rate of axillary metastasis and complications after axillary evaluation. Even though SLNB had lower rate of complications than ALND, its morbidity is not insignificant, with 41% of patients having upper extremity impairment at 7 years.^27^, ^28^ In this study, despite of a 51.98% upstaging rate, the rate of axillary metastasis is relatively low, which may support the omission of axillary evaluation in selected patients.

For DCIS patients, NCCN guideline recommended that axillary evaluation could be omitted in patients with BCS, whereas patients with mastectomy could receive SLNB considering primary operation may result in losing opportunity of future SLNB.^13^ For DCISM patients, axillary evaluation was still under debate. According to our study, patients with DCIS and DCISM on final pathology had similar metastasis rate, showing that the clinical meaning of upstaging from DCIS to DCISM is relatively limited. For IBC patients with clinically negative axillary, both NCCN and ASCO guidelines recommended SLNB.^12^, ^13^ However, in this study, 82.9% (160/193) of patients in IBC group had negative ALNs, 16.06% (31/193) had one to three positive ALNs, and only two patients had more than three positive ALNs. Interestingly, these two patients had the same histological type, both were invasive micropapillary carcinoma, which were proved to have worse prognosis than most histological types. Thus, axillary evaluation may also be omitted in IBC patients with low risk of axillary metastasis.

Certain measures should be taken place to reduce the upstage rate. First, we need to choose biopsy method carefully for these patients, in particularly some patients with calcification may be more suitable for vacuum‐assisted biopsy. Second, we should provide more choices in CNB, such as 8‐gauge CNB. The accuracy of preoperative diagnosis is in accordance with the quantity of samples. Third, the quantity of biopsy samples can be more individualized. According to this study, patients with larger tumor size on ultrasonography (>2.0 cm) are more likely to be upstaged on final pathology. Thus, biopsy samples should be taken more to improve the accuracy of their preoperative diagnosis.

According to our study, predictors of upstaging and axillary metastasis were not completely consistent, thus, the selective axillary evaluation for patients with a higher risk of upstaging may not accurately identify those with positive ALNs. After integrating predictors of upstaging and factors associated with positive ALNs in this study, we found that patients with larger tumor size (>2 cm), multifocal lesions or ER+HER2+ status were more likely to be upstaging patients with positive ALNs, who might need axillary evaluation more than patients with relatively low risk of upstaging or axillary metastasis. In previous studies, some authors also suggested that SLNB could be performed only for cases with high‐risk features, because the clinical benefit of SLNB needed to be balanced against the risk of complications.^29^, ^30^ In the future, we may able to further identify patients who can avoid unnecessary axillary evaluation based on their clinicopathological predictors, in conjunction with continued progress in adjuvant radiotherapy and systematic therapy which may be adequate to control axillary status in clinically negative patients.

There are some limitations to this study. First, this was a retrospective study. However, this was a relatively large dataset with uniform inclusive and exclusive criteria. Second, not all histological grades on CNB pathology were reported in our institution. While, according to previous studies, we believe that histological grade may be an important predictor of upstaging or axillary metastasis.^2^, ^8^, ^27^ Finally, the sample volume for preoperative pathological diagnosis were unknown. It is obvious that the accuracy of preoperative diagnosis is in accordance with the number of samples. Further assessment is needed to select patients with low risk of axillary metastasis, who can safely omit axillary evaluation.

## 5 CONCLUSION

Overall, our study demonstrated that, for patients diagnosed with DCIS by CNB, larger tumor size on ultrasonography (>2 cm) and molecular subtype (ER+HER2+) were two independent predictors of upstaging, whereas larger tumor size on pathology (>2 cm) and multifocal lesions were the strongest risk factors of axillary metastasis. Axillary evaluation was recommended in patients with larger tumor size (>2 cm), multifocal lesions or ER+HER2+ status. Despite of a 51.98% upstaging rate, the rate of axillary metastasis in these patients is relatively low, suggesting that axillary evaluation can be omitted in selected patients who have a low risk of upstaging or axillary metastasis.

## CONFLICT OF INTEREST

None.

## Data Availability

The data used to support the findings of this study are included within the article.
